# Molecular Testing on Cytology for Gene Fusion Detection

**DOI:** 10.3389/fmed.2021.643113

**Published:** 2021-07-06

**Authors:** Fernando Schmitt, Alessia Di Lorito, Philippe Vielh

**Affiliations:** ^1^Medical Faculty of Porto University, Porto, Portugal; ^2^Unit of Molecular Pathology of Institute of Molecular Pathology and Immunology of University of Porto, Porto, Portugal; ^3^CIntesis@RISE, Porto, Portugal; ^4^Department of Pathology, SS Annunziata Hospital, Chieti, Italy; ^5^Medipath & American Hospital of Paris, Paris, France

**Keywords:** NanoString, RT-PCR, fluorescence *in situ* hybridization, next-generation sequencing, gene rearrangements, cytology, gene fusions

## Abstract

Cytology samples are suitable for the study of genotypic and phenotypic changes observed in different tumors. Being a minimally invasive technique, cytology sampling has been used as an acceptable alternative to track the alterations associated with tumor progression. Although the detection of gene mutations is well-established on cytology, in the last few years, gene fusion detections are becoming mandatory, especially in some tumor types such as lung cancer. Different technologies are available such as immunocytochemistry, fluorescence *in situ* hybridization, reverse transcription-polymerase chain reaction, and massive parallel sequencing approaches. Considering that many new drugs targeted fusion proteins, cytological samples can be of use to detect gene fusions in solid and lymphoproliferative tumor patients. In this article, we revised the use of several techniques utilized to check gene fusions in cytological material.

## Introduction

In the last few years, targeted therapies have been revolutionary in cancer treatment. The discovery of new molecular alteration has allowed development and introduction of new technologies such as massive parallel sequencing (MPS) in clinical practice (1).

From a pathology point of view, this means the *need* to correlate tumor morphological features with immunophenotype and molecular aspects in order to treat each patient with the more appropriate drugs at the right time. The identification of a molecular target is also important to establish outcome and prognosis.

Cancer development is driven by different types of genetic alterations such as mutations, deletions, gene fusions, amplifications, and rearrangements. These alterations can be detected using different approaches in cells/tissue such as immunocytochemistry (ICC), immunohistochemistry (IHC), fluorescent *in situ* hybridization (FISH), reverse transcription-polymerase chain reaction (RT-PCR), and MPS using RNA-based and DNA-based approaches (2). The main advantages and disadvantages of these techniques are reported in Table 1.

**Table 1:** Fusion testing methods: advantages and disadvantages.

**Method**	**Advantages**	**Disadvantages**
ICC	- Rapid and low cost - Widely available	- Sensitivity depends on thebiomarkers antibody - Not easily multiplexed - Positive cases require orthogonal confirmation method for some biomarkers
FISH	- Established approach - Break apart FISH detects rearrangements without 5' partner knowledge	- High cost - Requires expert interpretation - Not easily multiplexed with other biomarkers - Not confirm detected fusionexpressed - Sensitivity and specificity depend on break apart assay utilized
RT-PCR	- Rapid and low cost - Well-established method	- Identify knowledge fusion partners - Might miss fusion due to break apart variability - Not confirmation that protein is present
MPS	- Possibility to study all clinically important genomic fusions - Most tissue sparing approach for broad genetic analysis - Commercially kit available - Detection of known and unknown fusions - Multiplexed	- Require high level of funding - High level bioinformatics ability - Large introns may be problematic (as for NTRK genes) for DNA-based MPS - Transcripts expressed at low levels by RNA-based can be a problem issue - Not confirmation that protein is present

*ICC, immunocytochemistry; FISH, fluorescent in situ hybridization; RT-PCR, Reverse-Transcription Polymerase Chain Reaction; MPS, massive parallel sequencing. For more details, see the text*.

An increasing interest is reported in this field especially in lung cancer. In the last 2 years, the US Food and Drug Administration granted accelerated approval or approval to specific fusion gene drugs in non-small-cell lung cancer (NSCLC) for *REarranged during Transfection proto-oncogene gene*/RET (selpercatinib), *ROS proto-oncogene 1*/ROS1 (alectinib or entrectinib), *anaplastic lymphoma receptor tyrosine kinase/*ALK (crizotinib, alectinib, etc.), and *neurotrophic tyrosine receptor kinase*/NTRK (entrectinib) fusions. Chromosomal rearrangements involving ALK, RET, ROS1, and NTRK family genes make in-frame kinase fusions with other partner genes causing uncontrolled proliferation of transformed neoplastic cells (3).

In lung cancer, patients are often diagnosed at the advanced stage of the disease, and they cannot benefit from primary tumor surgical resection. Small biopsies or cytology samples are more often the only pathologic specimens to guide systemic therapy. In literature, the utility of cytologic specimens in molecular testing has been demonstrated and validated in a lot of studies. For molecular analysis, although cytology samples provide high-quality material, application of molecular technologies on cytopathology is not yet widely used or recognized (2).

Indeed, in literature, many studies have demonstrated the use of cytology to check gene mutations, but little is known about detection of gene fusions in cytological samples (4, 5). In this review, we will discuss the new technological applications on routine cytological material for gene fusions.

## Methods

### Immunocytochemistry

ICC is widely applied in clinical practice to assess immunophenotype of neoplastic cells. It is a cost-effective and easily available technique, with rapid turnaround time, and can be applied on relatively few number of tumor cells. Compared to other molecular techniques, ICC has few technical challenges (6). Most predictive assays for biomarkers have been validated on formalin-fixed paraffin-embedded (FFPE) histologic tissue specimens. However, NSCLC patients frequently have diagnosis on cytology samples, and request for predictive biomarker testing on cytologic specimens is more frequently observed. Currently, there is no validation for biomarker detection on cytology, using ICC (7). Cytologic specimen preparations require a huge amount of critical preanalytic variables such as various collection media, fixatives, storage conditions, processing techniques, and stains, among others. Furthermore, ICC assays on the cytological samples need a rigorous and thorough validation process because FFPE histological tissue preparations have been used to validate and standardize all protocols (8).

Recently, the International Association for the Study of Lung Cancer (IASLC) Pathology Committee reported that “all cytologic preparations, including cell blocks, ethanol fixed, and air-dried slides” can be used for ICC” (9). Among the different cytologic sample preparations, cell blocks (CBs) are the most widely diffused and accepted. This is in part due to their availability in routine labs, the possibility of getting multiple sections to test a panel of markers, and the use of standardized and validated protocols for FFPE histologic tissue applicable to CBs with automated immunostainers. In fact, CB sections for ICC usually have a final fixation step prior to processing into an FFPE block utilizing 10% neutral buffered formalin. However, there are a great variability among cytopathology laboratories, and there are no standardized protocol for the prefixation, collection media to be used, and processing technique. Furthermore, it is important to note that CBs, as histological preparations, are formalin fixed, so for molecular analysis, they are associated with artifacts and loss of nucleic acid yield.

Non-CB cytologic preparations include air-dried and alcohol-fixed direct smears, cytospins, and liquid-based cytology (LBC) preparations and also require ICC validation and internal controls. Of these, cytospin and ethanol-fixed smear immunostainings are the most commonly utilized. ICC on previously stained slides can also be of use, usually after some formalin-based post-fixation step (9–11). In literature, authors suggested that some fixatives can alter antigenicity and ICC staining in cytologic specimens. On the other hand, the United Kingdom National External Quality Assessment Service (UK NEQAS) demonstrated that all non-formalin and formalin fixatives, with the exception of acetone, give comparable immunostaining quality (11, 12). In NSCLC, as reported by College of American Pathologists (CAP) guidelines, ICC can be of use to check ALK and ROS1 rearrangements. Recently, the NTRK IHC approach has been considered as a screening method to identify positive samples to be tested with orthogonal methods.

### ICC for ALK Rearrangements

ALK rearrangement testing, according to the current College of American Pathologist/International Association for the Study of Lung Cancer/Association for Molecular Pathology (CAP/IASLC/AMP) guidelines, requires FISH assay, PCR-based methods, or, alternatively, immunohistochemistry (IHC). ALK IHC can be performed using 5A4 (Novocastra, Leica Biosystems; Newcastle Upon Tyne, UK) and D5F3 (Cell Signaling Technology, Danvers, MA) clones.

The D5F3 ALK clone is used in a Ventana automated immunoassay (Ventana ALK D5F3 CDx Assay, Ventana Medical Systems, Tucson, AZ), approved by the United States Food and Drug Administration (FDA) as a companion diagnostic kit for crizotinib treatment in patients with ALK rearrangements^1^. A lot of studies regarding cytological samples used the D5F3 or 5A4 clones on CB preparations and demonstrated 100% sensitivity with specificities ranging between 83 and 100% (13–16).

LBC preparations, cytospin, and smears have been reported to have low sensitivity (66–100%), so ICC applied on non-FFPE samples needs more validation (17–19).

### ICC for ROS1 Rearrangements

For ROS1 rearrangements, the guidelines recommended to use, as a screening tool, D4D6 (Cell Signaling Technology) clone that is reported to be highly sensitive but relatively less specific. Therefore, IHC-positive cases require cytogenetic or molecular test confirmation (20). Unlike ALK, FDA has not approved ROS1 IHC using D4D6 as a companion diagnostic for patients to be tested for ROS1 rearrangements. There is no specific cutoff of positivity for ROS1 IHC evaluation because of some difficulties of interpretation. ROS1 protein can be expressed in non-neoplastic cells especially in hyperplastic type 2 pneumocytes, alveolar epithelial and basal cells, bronchial epithelial and metaplastic bronchiolar cells, and peribronchial glands. Indeed, positivity intensity has been found to be variable both in cell lines and in *ROS1*-rearranged cancers. Heterogeneous staining patterns (strongly positive and negative areas within the same tumor) is not common in *ROS1 positive* carcinomas. Some authors have proposed a ≥2 intensity of cytoplasmic staining in 50–75% of tumor cells or H-scores of 100–150 to reach high sensitivity and specificity. A positive sample on IHC should be processed for confirmatory testing as FISH or RT-PCR or MPS.

Regarding cytological material, some ICC studies of ROS1 D4D6 have been reported in literature. CBs as well as non-CB specimen immunostaining has shown sensitivities of 88–100% and specificities of 92–98% (21). In another study, ICC using the D4D6 antibody on an automated immunostainer was used on cytological specimens in the routine diagnostic setting. ROS1 ICC was tested on 295 NSCLC patients. The sensitivity, specificity, and positive and negative predictive values for ROS1 ICC compared with the final ROS1 status all were 100% (22).

Another ROS1 antibody (SP384) has been developed and studies reported high sensitivity and specificity, but data on cytology samples are not available (23).

### ICC for RET Rearrangements

In NSCLC, chromosomal rearrangements involving RET gene represent a small percentage of patients (1–2%). At the beginning, RET rearrangements in NSCLC works have been reported to be more common among never-smokers with the adenocarcinoma histologic subtype. Retrospective consequent analysis demonstrated that patients with RET rearrangements had significantly more poorly differentiated carcinomas compared with ALK positive patients. Very recently, thyrosine kinase inhibitors as Selpercatinib and Vandetanib have been proven to be efficacious in RET-positive NSCLC patients. Regarding the possibility to use IHC, in research, the most used clone is EPR2871 antibody. Yang et al. showed that the sensitivity of IHC depends on the fusion partner. *KIF5B* sensitivity was highest (100%), followed by *CCDC6* (88.9%) and *NCOA4* (50%). RET IHC specificity was 82% (24). No reports are published, yet, about ICC.

### ICC for NTRK Rearrangements

Recently, NTRK family gene novel fusions have been described in a subset of tumors. Chromosomal translocations involving NTRK1, NTRK2, and NTRK3 genes cause a constitutive activation and aberrant expression of TRK kinases in a series of different cancer types. NTRK alterations are very rare in most common malignancies, ranging between 0.1 and 2% according to the tumor type. A selective neurotrophic tyrosine receptor kinase (NTRK) inhibitor, larotrectinib, has been approved for NTRK-positive patients by FDA.

In NSCLC patients, these fusions occur in a very small group of patients (<1%) (25). Different IHC antibodies have been evaluated in literature and used as screening tool (26, 27). In fact, there are antibodies against NTRK proteins (Trk-A or Trk-B), antibodies against common amino acid sequences, found in all Trk proteins (pan-Trk antibodies), and antibody cocktails. The most well-studied and reported clone is the pan-Trk antibody EPR17341 (Abcam and Roche/Ventana). The antibody recognizes a homologous region of Trk-A, Trk-B, and Trk-C near the C-terminus (28). In lung carcinomas, NTRK IHC using the EPR17341 clone shows a sensitivity of 87.5% and a specificity of 100%. However, the positivity, confirmed by second technology, is very rare, accounting for <1% (28). The staining intensity is variable; most studies reported diffuse positivity in neoplastic cells, but for some authors, a case can be considered positive with at least 1% of positivity. Indeed, the immunohistochemical staining pattern has been reported to correlate with a specific genic rearrangement and fusion partner. Furthermore, IHC seems to have higher specificity in some tumor histotypes in lung, colon, and thyroid and less specificity for NTRK3 fusions. Recently, the European Society for Medical Oncology (ESMO) proposed an algorithm for the NTRK fusion detection. Strong and diffuse cytoplasmic staining should be considered a surrogate of NTRK1/NTRK2 fusions while nuclear positivity should be a surrogate of NTRK3 fusions. Molecular testing is required for confirmation for weak cytoplasmic staining neoplasm (29).

Regarding cytological samples, these biomarkers have been tested on histological samples, and cytological specimens were excluded from clinical studies. CBs, however, could be of use for studying NTRK molecular status. Alcohol-fixed direct smears have not been used for NTRK ICC. Furthermore, direct smears can be utilized for DNA extraction for molecular approaches as targeted NGS. However, at the present time, there are no studies published about the use of cytological samples for NTRK fusions, detected by ICC, FISH, and MPS.

### Fluorescence *in situ* Hybridization

The FISH assay is widely applied to detect rearrangements, using dual-labeled, break-apart probes. This technique does not require an a priori knowledge of the fusion partner. Direct smears or CBs or LBC samples are currently being used for FISH assays. Nuclei on direct smears are well-preserved, which allows the true number of FISH signal detection on neoplastic cells and avoids signal loss from truncation artifacts.

FISH is considered the gold standard for a lot of cases and can easily find amplification, rearrangements, and polysomy. However, it does not identify the exact fusion variant. Although multiplexing analysis has been reported, FISH usually requires a single test for each gene to be analyzed.

Of note, break-apart probes cannot identify small intrachromosomal rearrangements, and not all known DNA rearrangements produce an expressed fusion transcript.

This is widely reported especially for EML4-ALK variants of fusion (30, 31).

**Figure 1:**
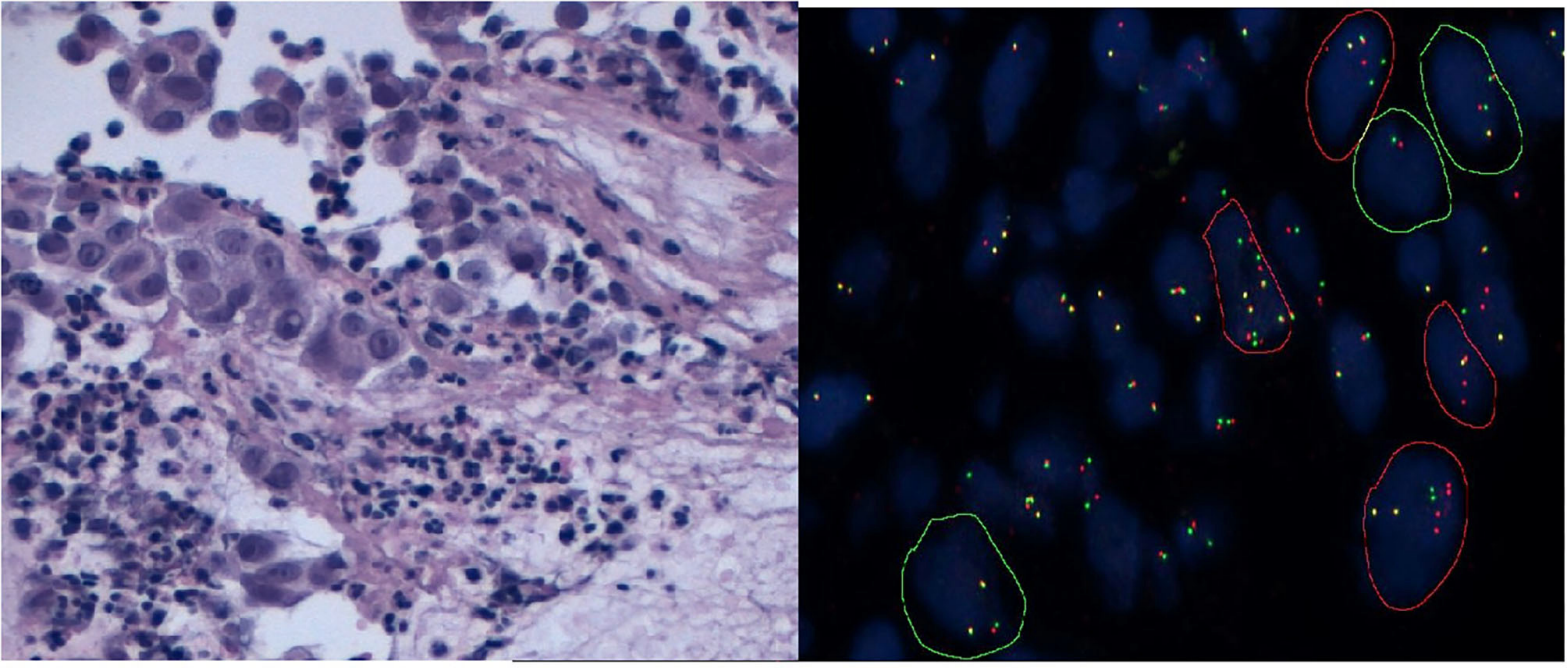
An ALK-positive case detected on lung adenocarcinoma cell block. H&E staining and FISH.

In NSCLC, for ALK or ROS1 or RET fusions, FISH testing has long been considered a gold standard. FISH is positive when rearrangement is present in at least 15% of cancer cells. In ALK rearrangement positive specimens, nuclei show “broken-apart” red and green signals, which are separated by at least two signal diameters. However, if there is ALK deletion, nuclei will show single red signals (Figure 1). Normal cells have yellow, intact signals.

**Figure 2:**
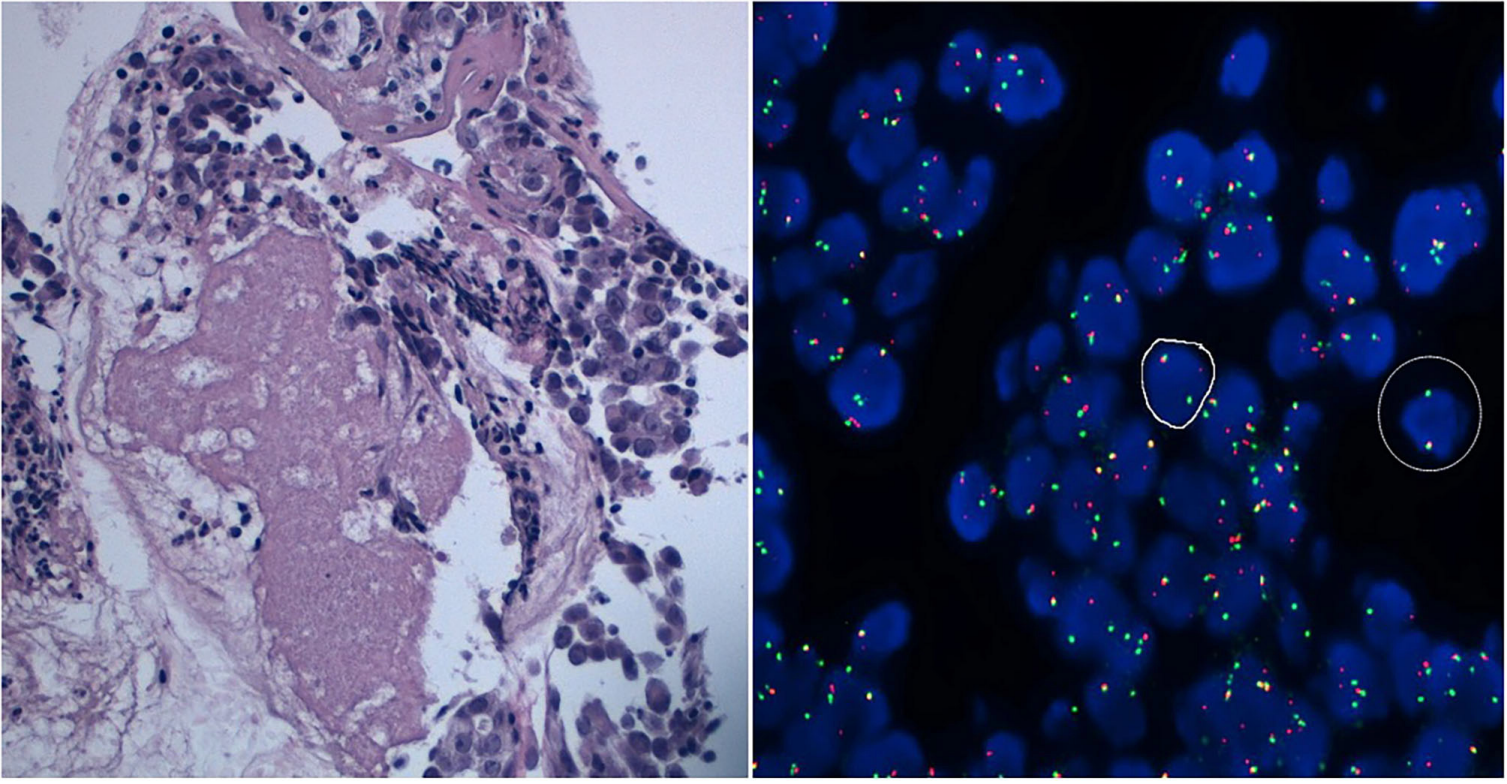
A ROS1-positive case detected by FISH on lung adenocarcinoma cell block. H&E and FISH.

In specimens positive for ROS1 and RET, there is either the classical pattern—one fusion signal (native ROS1/RET) and broken-apart green and red signals—or the atypical pattern—one fusion signal (native ROS1/RET) and one green signal without red signal (Figure 2). Regarding NTRKs, it is necessary to study with distinguishing probes NTRK1, NTRK2, and NTRK3. This requires more samples, considering that multiplexed FISH is not possible at the present time and requires >1 assay to cover all NTRK gene fusions. As for the other genes described, a case is considered as positive with more than 15% of split signals.

FISH analysis can be performed on CB, Diff-Quik, and Papanicolaou-stained smears, and ThinPrep slides. CBs have been widely used for ALK/ROS1 rearrangement analysis, because the histological protocol can also be validated on this material. Ethanol-based fixation of cytologic smears is also feasible for FISH assay. For the analysis, the selection of an area of the smear where cells did not overlap is important. In this way, entire and individual nuclei can be analyzed; areas of nuclear debris and overlapped nuclei should be avoided. To assess FISH signals, at least 100 tumor cells with entire nuclei on a monolayered area should be considered. An automated or semi-automated platform with dedicated software for analysis should be encouraged (32).

Zito Marino et al. described the use of multiplex FISH with simultaneous *ALK* rearrangement and *ROS1* rearrangement analysis on a single slide, using cytology material (33). It is reported that dual ALK/ROS1 FISH probe test results were fully concordant with the results of previous single FISH tests of ALK/ROS1 on two different slides, without false negativity and false positivity. Indeed, multiplexed ALK/ROS1 FISH test showed agreement with IHC (33).

However, FISH requires expertise and is relatively costly and time-consuming, leading to a long turnaround time. Moreover, the advanced equipment necessary for this procedure is not available in all laboratories.

Indeed, the performance of FISH depends on some preanalytical factors such as time of fixation and fixative types. It should be noted that false-positive and false-negative FISH results, even by experienced laboratories with microscope equipped with dedicated software program such as the FDA-approved BioView scoring system (Abbott Molecular, Abbott Park, Illinois), have been reported (34). Currently, a positive cell rate near 15%, which is considered “borderline,” needs additional analysis, especially in young patients (35, 36).

### Reverse Transcription-Polymerase Chain Reaction

RT-PCR is a high-specificity technique that uses specific primers to check fusion transcripts at RNA levels. Primer pairs specific for the known fusion are necessary for the investigation and high-integrity RNA obtained following an immediate extraction of nucleic acid from fresh and unfixed material. It is known that RT-PCR results may not always be informative when RNA is extracted from FFPE samples such as CBs. Archival cytological slides and brush material can be used for RNA direct extraction. However, the RNA quality depends on inadequate fixation or prolonged tissue ischemia, and these preanalytical factors can cause RNA degradation.

RT-PCR assays, even when multiplexed, cover only the most common fusion variants and those that the assay was designed to identify, missing all the unknown variants (37).

This is particularly true in NSCLC where ALK and ROS1 rearrangements occur with known and unknown gene partners, leading to a lot of fusion variants, so that RT-PCR likely misses rare variants (38). Furthermore, the possibility of evaluating the 3'−5' imbalance of these genes should overcome it as reported in literature by the European Thoracic Oncology Platform Lungscape Project work (39). One of the kits available for ALK detection is the ALK RGQ RT-PCR test (QIAGEN Manchester, UK), while for ROS1 rearrangements, some authors utilized the QIAGEN OneStep RT-PCR kit (Qiagen, Hilden, Germany). The use of RT-PCR for NTRK rearrangement detection has been reported in thyroid carcinoma, salivary and breast secretory carcinoma, congenital fibrosarcoma, and glioblastomas. However, the variability and the complexity of these rearrangements together with previously described limits restricted its use in clinical settings.

The current approach and guidelines suggest not to use RT-PCR as an alternative to IHC or FISH, but as an additional method in the rare discordant IHC and FISH cases (7).

### nCounter System—NanoString Technologies

An alternative method to detect fusion is the nCounter system (NanoString Technologies). It is a fast hybridization method that uses low RNA quantity to study gene fusions. Each target of interest is detected by a unique pair of reporter and capture probes whose sequences are adjacent and complementary to a specific RNA messenger. The reporter probe is linked to a digital color barcode and the capture probe is biotinylated. In a multiplex reaction, each pair of probes is hybridized with the targeted messenger RNA. After the immobilization of the hybridized complexes, a phase of elongation occurs to enable the detection and then counting of specific fluorescence barcodes is made (40).

NanoString gene fusion analysis is based on a dual evaluation: assessment of the 30- and 50-gene region imbalance and transcripts using target-specific probes, and detection of known fusion. This system shows a high specificity and sensitivity, with a good throughput, analyzing up to 800 targets for 12 samples simultaneously.

In NSCLC, it is possible to detect at the same time, in a few working days, ALK, ROS1, RET, BRAF, and MET proto-oncogene (MET)-skipping transcripts (41).

Most analyses using the NanoString method have been made on histological specimens; however, it may be well-applied also on cytological ones as suggested by Sgariglia et al. (42).

Data from literature reported high concordance between nCounter RNA gene fusion assay results and IHC, FISH, and RT-PCR in NSCLC cases (43–45).

### Massive Parallel Sequencing

MPS leads the study of multiple mutations in multiple genes in different patient specimens in a single run. In the recent past, the high costs of this technology limited its development and it was feasible only in dedicated large diagnostic centers. However, the commercially available targeted panels (allowing to sequence specific areas of the genome and detecting known and novel variants within the region of interest) with dedicated automated bioinformatics pipelines lead the diffusion of MPS assays (46). In fact, in the past, MPS was mainly used in research to study the comprehensive whole genome or transcriptome, while, recently, the use of targeted panels with limited numbers of gene of interest to sequence has brought it into clinical practice. It is also possible to discriminate point mutations, insertions, deletions, and copy number variations at the same time. In the case of fusion gene, it detects known and unknown rearrangements. This possibility is important for cytological material with limited amount of neoplastic cells. Regardless of the specific features of platform used, the gene fusion detection workflow has sequential phases: DNA library construction, single-fragment clonal amplification, MPS, and sequencing data analysis with the informatics pipeline. The most widely used MPS assays are the Illumina platforms (San Diego, California), the IonTorrent series (Thermo Fisher Scientific, South San Francisco, California), and the Qiagen assay (GeneReader, Qiagen, Hilden, Germany). They are different for run time, DNA/RNA input requirements, panels available, target enrichment, sequencing chemistry, and cost (47).

In clinical routine, gene fusions can be analyzed by MPS at DNA or RNA levels, and in both cases, targeted panels are considered better than non-targeted, largest gene panels. Furthermore, some panels are commercially available, some of which are approved for diagnostic use or can be customized. Almost all panels analyzed in literature have a good agreement with other methods such FISH and IHC (31, 48, 49).

Cytological samples can be used to detect deletions, point mutations, and gene rearrangements by MPS. Several studies demonstrated the possibility to extract high-quality RNA for MPS analysis. In a study, Velizheva et al. showed the feasibility of non-formalin cytology specimens for the simultaneous MPS testing of lung adenocarcinomas by amplicon resequencing panels. Using direct smears for RNA-based MPS analysis, they reported high sensitivity (100% for DNA and RNA) and specificity (96 and 100% for DNA and RNA) (50).

There are different approaches to study gene fusion: RNA and/or DNA approaches.

RNA-based assay detects only expressed fusion genes and can discriminate splicing isoforms, with a quantification of fusion transcripts. RNA sequencing is not affected by intronic regions but RNA extraction is more complicated than DNA, especially purification from FFPE specimens, as it can be highly degraded with the possibility to invalidate the run (50). However, in this setting, cytological samples as direct smears instead of CB preparation improve the adequacy of cytological material for RNA fusion testing for predictive biomarkers, as reported (51).

DNA-based assay does not need an additional RNA purification step and allows the detection of the exact gene fusion breakpoints together with other alterations such as single nucleotide variants, indels, copy number variations, and duplication. However, the evaluation of the rearranged locus expression is not possible. Furthermore, the sequencing results can be affected by detection of some fusion events involving intronic regions, which can be extremely large with repetitive sequences. This is particularly true for some genes such as NTRK2 and NTRK3.

The possibility to use targeted panels in clinical practice has been revolutionary. It is faster, it requires a lower input of starting material, data analysis and result interpretation are not so difficult, and analysis is based on a limited number of clinical valuable targets.

Another important point is that DNA- or RNA-targeted panels for gene fusions are amplicon-based and hybrid-capture. The hybrid-capture technique needs a gene-specific enrichment by a hybridization step with probes complementary to the regions of interest. The amplicon-based approach utilized primers specific for each target, depending on a multiplex PCR (mPCR). For gene fusion detection, the amplicon-based method is one of the most common technique and different commercially available and custom panels have already been reported and validated.

Classical mPCR allows the discovery of known fusion variants, and it is based on the use of primers with exon–exon fusion combinations. Indeed, RNA-based fusion panels also include testing for expression imbalances between 50 and 30 regions of the target genes. In this way, it allows rearrangement identification even if the fusion partner is not included in the panel or unknown. Study of comparison with FISH, IHC, and RT-PCR showed that this test has a good concordance and in some cases >90%. In general, for gene fusion analysis, amplicon-based assay needs RNA purification and implies the use of combined DNA and RNA NGS tests. The main problem is that, in some cases, it could be difficult to separate tissue sections and obtain enough material for analysis (52).

The hybrid-capture approach requires a gene-specific enrichment by a hybridization step with biotinylated DNA or RNA probes specific for the regions of interest. After DNA extraction, probes are complementary to intronic, exonic, and intergenic regions, whereas if RNA is analyzed, probes target only exonic regions. This method allows the identification of known and novel fusion variants for any gene targeted by the capture panel. Novel fusion genes are not discovered, even if at least one of the fusion partners has to be present on the target panel. There are commercially hybrid-capture panels specific for RNA available from Illumina (i.e., Trusight RNA fusion panel) and Agilent (i.e., SureSelect all-in One Solid tumor).

In the setting of gene fusion analysis, DNA hybrid-capture panels are more common than the RNA ones. At this moment, in clinical practice, there are two DNA hybrid-capture panels approved by the FDA: the Memorial Sloan Kettering (MSK) Integrated Mutation Profiling of Actionable Cancer Targets (IMPACT) and the FoundationOne CDx—Foundation Medicine (Roche). These panels allow one to study mutations, copy number alterations, and rearrangements in 468 and 324 cancer-associated genes, respectively, besides the evaluation of tumor mutational burden (TMB) and microsatellite instability (MSI). In comparison to FoundationOne CDx, the MSK-IMPACT panel needs the simultaneous analysis of tumor and normal DNA. Other smaller DNA-targeted panels have been analyzed on clinical samples with good sensitivity and specificity and requiring 50–250 ng of DNA input (52).

Furthermore, this approach has also been specifically applied on some clinical samples such as Endobronchial Ultrasound Guided Transbronchial Needle Aspiration (EBUS). In a recent study, Xie et al. evaluated 85 EBUS specimens using the Lung Core 56 gene panel (Burning Rock Biotech; Asia-Pacific). They found 77 samples to be adequate when the amount of tumor cells was very low (5%) (53). In an another study, Ruan et al. (54) analyzed 108 malignant effusions of lung cancer patients. They described the use of a panel including 17 lung cancer-associated genes, and they successfully identified both gene rearrangements and mutations.

The gene fusion analysis at the DNA level offers important advantages: DNA is more stable than RNA and a unique NGS test can allow a complete tumor molecular characterization. Sensitivity of DNA-based NGS, however, is lower if fusion breakpoints involve long intronic regions that are not covered by hybridization-capture probes.

In general, for gene fusion analysis, the MPS approach allows more target analysis with sparing material with an acceptable turnaround time. It has a great sensitivity regardless of the material samples utilized (cytology, plasma, biopsies, surgical, or fresh samples), but it needs an appropriate validation procedure and protocol optimization, available interpretive software and bioinformatic support, and worker expertise. Finally, MPS allows incorporation of newly discovered biomarkers in the clinical practice, and for gene fusion analysis, this is of great importance (55).

## Conclusions

In summary, whether gene fusion analysis is done individually or in a panel will lead to better prognostic and therapeutic stratification in NSCLC patients in a routine clinical setting. Cytological sampling is an appropriate first approach to have material in small lesions and/or in cancer patients unsuitable for surgical procedures (56). Although cytology shows intrinsic limitations, the possibility to obtain CB from cytological material may enhance the diagnostic cytology rate and allow the application of ancillary techniques, validated on FFPE samples, to study predictive biomarkers (57). Different technologies are available in studying gene fusions such as ICC or RT-PCR or FISH and MPS approaches. Considering that many new drugs targeted fusion proteins, cytological samples can be of use to detect gene fusions not only in lung carcinomas but also in other solid and lymphoproliferative tumor patients.

## Author Contributions

FS: contributed to the study conception, design, and final review of the manuscript. ADL: written the first draft supervised by FS and contributed with the figures. PV: final review of the manuscript. All authors contributed to the article and approved submitted version.

## Conflict of Interest

The authors declare that the research was conducted in the absence of any commercial or financial relationships that could be construed as a potential conflict of interest.

## Footnotes

^1^https://www.fda.gov/drugs/resources-information-approved-drugs/fda-approves-crizotinib-capsules.
